# Assessment of Bovine Trypanosomiasis and Tsetse Fly Density in Gechi District, Western Ethiopia

**DOI:** 10.1155/japr/5512514

**Published:** 2025-08-14

**Authors:** Gebremedhin Gebrezgabiher, Kelifa Jemal Siraj, Gebremedhin Romha

**Affiliations:** ^1^Department of Veterinary Medicine, Samara University, Samara, Afar, Ethiopia; ^2^Department of Veterinary Public Health and Food Safety, Mekelle University, Mekelle, Tigray, Ethiopia

**Keywords:** bovine trypanosomiasis, Ethiopia, Gechi District, tsetse density

## Abstract

**Background:** Bovine trypanosomiasis, a parasitic disease transmitted by tsetse flies, poses a significant threat to livestock health and agricultural productivity in Ethiopia, affecting millions of farmers who rely on cattle for milk, meat, and draught power. This study was aimed at assessing the prevalence of bovine trypanosomiasis and the density of tsetse flies in the Gechi District, western Ethiopia.

**Methods:** A cross-sectional study was conducted from March to September 2022 in Gechi District, western Ethiopia.

**Results:** Out of 471 blood samples collected from randomly selected animals, 32 (6.79%) tested positive for trypanosomes, with *Trypanosome vivax* (71.87%) being the most prevalent, followed by *Trypanosome congolense* (25%) and *Trypanosome brucei* (3.13%). The prevalence of trypanosomes did not significantly differ across study sites, age groups, body conditions, or sexes. The mean packed cell volume value of parasitemic animals (23.1%) was significantly lower than that of aparasitemic animals (25.52%) (*p* = 0.013). By deploying 60 traps, a total of 288 tsetse flies were caught, with 73.26% identified as *Glossina tachinoides* and the remaining 26.74% as *Glossina morsitans*.

**Conclusions:** This study found a 6.79% prevalence of bovine trypanosomiasis in the Gechi District, which was caused primarily by *T. vivax* and *T. congolense*, with a tsetse fly density of 2.4 flies per trap per day (FTD), indicating that trypanosomes and their vectors are prevalent in the area. Continuous control measures and monitoring should be implemented to mitigate the impact of the disease.

## 1. Background

Bovine trypanosomiasis, a parasitic disease caused by protozoa of the genus *Trypanosoma*, poses a significant threat to livestock health and agricultural productivity in sub-Saharan Africa [1–3]. The disease is transmitted primarily through the bites of infected tsetse flies (*Glossina* species) [4]. Additionally, it can also be mechanically spread through the activities of hematophagous flies, such as *Stomoxys* and *Tabanus* species [5]. It manifests through various symptoms, including fever, weakness, anemia, immunosuppression, and tissue damage, which can lead to severe cases and even death [3]. Beyond affecting cattle health and productivity, bovine trypanosomiasis also hampers the overall growth of the agricultural sector in affected areas [6, 7].

Bovine trypanosomiasis poses a significant threat to livestock health in Ethiopia, impacting agricultural output and affecting the livelihoods of farmers [8]. The primary trypanosome species responsible are *Trypanosoma vivax*, *Trypanosoma congolense*, and *Trypanosoma brucei* [9]. The country's extensive agricultural landscape and major river basins, especially in the west, southwest, and northwest, create an ideal environment for tsetse fly breeding [10], rendering up to 200,000 km^2^ of arable land unsuitable for agriculture [3]. Historically, six species of tsetse flies have been recorded in Ethiopia: *Glossina pallidipes*, *Glossina tachinoides*, *Glossina morsitans*, *Glossina fuscipes*, *Glossina longipennis*, and *Glossina brevipalpis* [11].

Previous studies conducted in Gechi and other districts within the Buno Bedele Zone, as well as neighboring areas, have documented varying prevalence rates of bovine trypanosomiasis and tsetse fly populations [8, 10, 12, 13]. However, the distribution of trypanosomes and tsetse fly populations is continually shifting due to a range of factors, such as ecological disturbances, climate change, and human activities [14]. Thus, the presence of these vectors and the disease burden, as well as associated risk factors, necessitate continuous updating and monitoring to implement control measures and mitigate their impact on livestock production [9]. This study was undertaken to assess the prevalence, associated risk factors of bovine trypanosomiasis, and the apparent density of its vectors in the Gechi District, western Ethiopia. By understanding the distribution and density of tsetse flies, as well as the prevalence of trypanosome infections, effective strategies can be developed to control and reduce the incidence of this debilitating livestock disease.

## 2. Materials and Methods

### 2.1. Study Area

**Figure 1:**
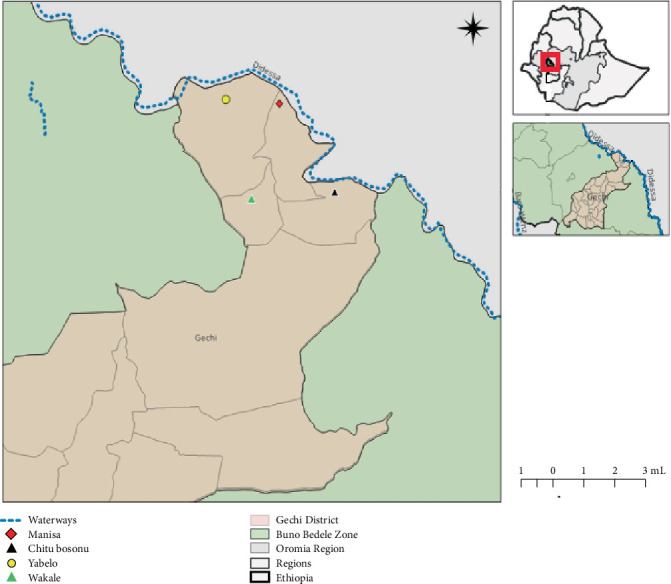
Map of Gechi District and areas where study animals were sampled.

The study was conducted in Gechi District of Buno Bedele Zone of Oromia region, western Ethiopia. It is located approximately 460 km from Addis Ababa at latitudes 8°16⁣′60⁣^″^ and longitudes 36°39⁣′59⁣^″^ (Figure 1). Gechi is bordered to the south by Didessa, to the north by Bedele, and to the east by the Jimma zone, with the Didessa River forming the eastern boundary. The district covers 1140.57 km^2^, with altitudes ranging from 1400 to 2010 m above sea level. The annual mean temperature varies from 12.5°C to 27.5°C, and the area receives over 1400 mm of rainfall annually. The vegetation type of the district is predominantly characterized by agroforestry such as coffee-based agroforestry, fruit tree–based agroforestry, and woodlots. Additionally, the district features natural vegetation, such as forests and grasslands. The Didessa River supports riverine forests, while savannah dominates upland areas.

The farming system is predominantly mixed, including crop production and livestock rearing. Farmers grow key crops, including teff, maize, sorghum, sesame, and tiger millet, alongside cash crops like coffee, which is a significant source of income in the region. Livestock, including cattle, sheep, and goats, play a vital role in providing food, income, and draft power for agricultural activities. The district is home to a population of 76,122 cattle [15]. Crop and livestock sales are vital income sources, with poorer groups also engaging in agricultural labor and firewood sales. Livestock husbandry and management in the district include communal grazing on natural pastures during the wet season, supplemented with grain residues. Livestock are sheltered in open kraals or nearby structures for protection against predators and harsh weather. For the purpose of this study, four peasant associations, namely, Chitu bosonu, Manisa, Yabelo, and Wakale, were purposively chosen on the basis of information from the district's office of livestock and fishery.

### 2.2. Study Design and Period

A cross-sectional study was conducted from March to September 2022 to assess the prevalence of bovine trypanosomiasis and apparent density of its vectors in four peasant associations within the study district.

### 2.3. Study Animals

The study involved zebu cattle of various sexes, age groups, and body conditions, managed under smallholder mixed crop-livestock farming systems.

### 2.4. Sample Size Determination

The number of animals required for the study was determined using Thrusfield's [16] formula:

 $$\begin{array}{c} N=\frac{1.96^{2}\times P_{\exp}\times \left(1-P_{\exp}\right)}{d^{2}}, \end{array}$$

where *N* is the required sample size, *P*_ex*p*_ is the expected prevalence, and *d* is the desired absolute precision.

Using a 95% confidence level, an expected prevalence of 3.4%, and a desired absolute precision of 0.05%, the sample size was calculated to be 51. However, to increase the precision of the study by a factor of *k*, this sample size was multiplied by *k*^2^ [17]. In our case, we took *k* = 3, and the calculated sample size was 459. Lastly, a total of 471 animals were included. The animals were randomly selected from the four peasant associations, which were purposively chosen on the basis of information from the district's office of livestock and fishery. The distribution of the animals was as follows: 136 from Chitu bosonu, 130 from Manisa, 143 from Yabelo, and 62 from Wakale.

### 2.5. Sampling Strategy

A systematic random sampling technique was used to select cattle for blood sampling. In each village, the sampling frame consisted of the total number of cattle present at the communal grazing points, provided by the local peasant associations. A sampling interval (*k*) was calculated by dividing the total number of cattle in the sampling frame by the required number of samples (*k* = *N*/*n*). The first animal was selected randomly by drawing a number between 1 and *k*, and subsequent animals were chosen at regular intervals (every *k*^th^ animal) thereafter. Care was taken to ensure that the selection process did not coincide with any observable cyclical patterns in herd grouping or order of arrival, which could bias the representativeness of the sample. Farmers then assisted in restraining the selected animals for blood sampling. During the sampling process, the sex, age, and body condition of the animals were recorded. The body condition score for each cattle was estimated using a technique involving visual inspection and palpation, categorizing the animals as good, medium, or poor based on the amount of fat covering the rump and loin, as well as the degree of depression in the tail head area [18]. The age of the animals was estimated as described by Pasquini and Spurgeon [19] and supplemented by information provided by the owners. Briefly, dentition was examined, with cattle having deciduous teeth and erupted central incisors classified as young (less than 2 years old) and those with erupted first, second, and corner incisors, as well as noticeable wear on the incisors classified as adults (above 2 years old).

### 2.6. Study Methods and Procedures

#### 2.6.1. Parasitological Survey

Paired blood samples were aseptically collected into heparinized microhematocrit tubes by puncturing the margin of the ear vein with a lancet and then transported to Bedele Regional Veterinary Laboratory for examination. One end of each capillary tube was sealed with sealant (Hawksley Ltd., Lancing, United Kingdom) and centrifuged at 12,000 rpm for 5 min to concentrate trypanosomes in the buffy coat and separate blood cells. The first capillary tube was used to determine the packed cell volume (PCV), which was then read on a hematocrit reader and recorded. The second capillary tube was broken 1 mm below the buffy coat, expressed onto a microscopic slide, mixed, and covered with a 22 × 22 mm cover slip. The slide was examined under a 40x objective using the dark ground buffy coat technique to detect the presence of motile trypanosomes. For positive samples, thin blood smears were prepared by placing a drop of blood near one end of a microscope slide. Another slide was held at a 30°–45° angle, and the edge of the spreader slide was gently touched to the blood drop, allowing the blood to spread along its edge. The spreader slide was then smoothly and quickly pushed across the surface of the first slide to create a thin smear. The smear was allowed to air dry completely, fixed with methanol for 5 min, stained with Giemsa stain, and examined under oil immersion at 100x magnification to identify the trypanosome species [20].

#### 2.6.2. Entomological Survey

**Figure 2:**
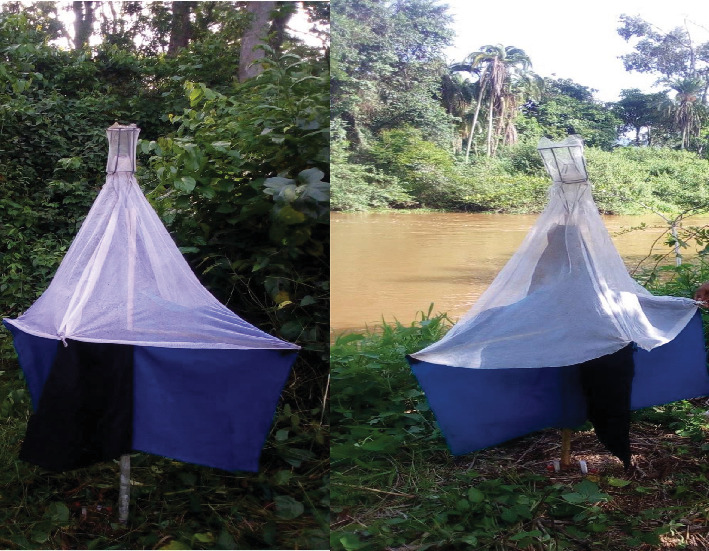
A baited monoconical trap deployed in suitable tsetse habitat (riverbank) in Gechi District.

**Figure 3:**
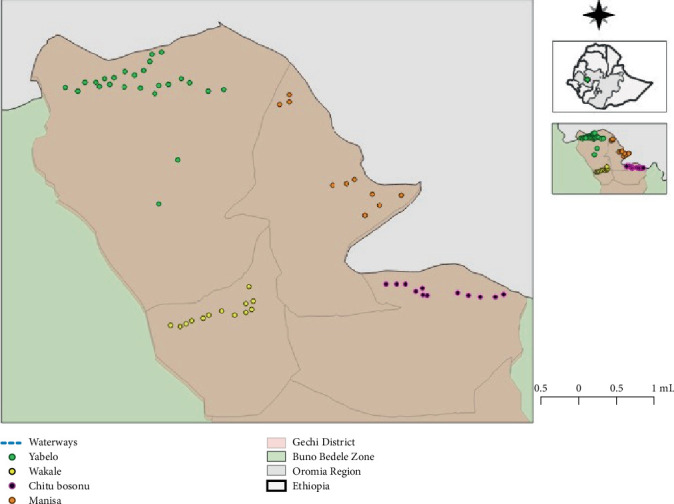
Map of the study area showing different fly trapping sites in four peasant associations in Gechi District.

A total of 60 baited monoconical traps (Figure 2) were deployed in selected suitable tsetse habitats (Figure 3), such as riverbanks and savannah lands, to assess the apparent densities, distributions, and species of tsetse flies involved in the transmission of trypanosomes. The monoconical traps were chosen due to their availability and proven effectiveness for both riverine and savannah tsetse species in similar agroecological settings [21, 22]. Each trap was baited with acetone, octanol, and cow urine in three separate bottles and placed at intervals of 200–250 m [23–25] depending on the surrounding vegetation [26]. To prevent the capture of flies by insects such as ants, the base of each trap pole was smeared with grease. Moreover, the geographic coordinates of trapping sites were recorded with a Global Positioning System. After 48 h, the cages were collected, and the captured flies were identified on the basis of their habitat and morphological characteristics or keys [27] (File S1). The tsetse flies were sexed by observing the posterior end of the ventral aspect of the abdomen via a hand lens. Male flies were identified by their enlarged hypopygium at the posterior ventral end of the abdomen. The arithmetic mean catch per trap per day (apparent density) of the tsetse flies was calculated by dividing the total number of tsetse flies (*F*) caught by the product of the number of traps used to catch them (*T*) and the number of days for which the traps were operational (*D*): *FTD* = Σ*F*/*T*∗*D*  [28, 29].

### 2.7. Data Analysis

The raw data were entered into Microsoft Excel 2016. Data analysis was performed via SPSS (Statistical Package for Social Science) Version 20 software, and descriptive statistics were employed to summarize the data. Prevalence was calculated as the number of infected individuals divided by the number of individuals examined, multiplied by 100. The associations between the prevalence of trypanosome infection and different variables, such as age, sex, and body condition, were assessed via univariate and multivariate binary logistic regression. PCV values for parasitemic and aparasitemic cattle were calculated. The mean PCV values between parasitemic and aparasitemic cattle were compared via an independent sample *t* test. A *p* value of less than 0.05 at a 95% confidence interval was considered statistically significant.

## 3. Results

### 3.1. Parasitological Findings

**Figure 4:**
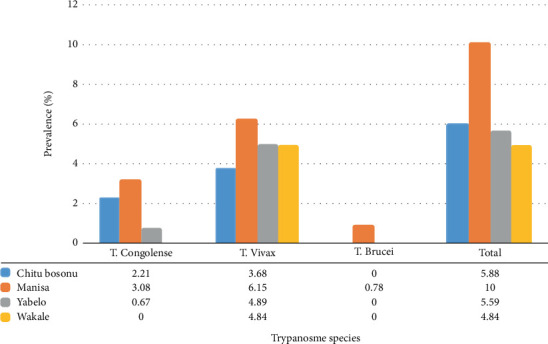
Prevalence of trypanosome species at four peasant associations in Gechi District, western Ethiopia (2022).

A total of 471 animals were sampled, including 136, 130, 143, and 62 from Chitu bosonu, Manisa, Yabelo, and Wakale peasant associations, respectively. Among these, 32 animals tested positive for trypanosomes, resulting in an overall bovine trypanosomiasis prevalence of 6.79% in the study area. At the peasant association level, the prevalence of infection was 5.88%, 10.0%, 5.59%, and 4.84% in the Chitu bosonu, Manisa, Yabelo, and Wakale peasant associations, respectively. Additionally, three species of trypanosomes were identified: *T. vivax* (71.87%), *T. congolense* (25%), and *T. brucei* (3.13%). The prevalence and distribution of the three trypanosome species across the four peasant associations in the study district are depicted in Table 1 and Figure 4.

### 3.2. Risk Factors for Bovine Trypanosomiasis

Table 2 presents the results from both univariate and multivariate logistic regression analyses examining the associations between different risk factors and trypanosome infection status. Although the associations among the considered variables were not statistically significant, the overall prevalence of infection was higher among female cattle (7.10%), adult cattle (7.21%), and cattle with poor body condition (13.33%).

### 3.3. Hematological Findings

The PCV of individual animals was measured to assess the degree of anemia. Among the examined cattle, 40.55% (191/471) were anemic. Moreover, the mean PCV of parasitemic cattle (23.1 ± 5.21) was significantly lower than the mean PCV of aparasitemic cattle (25.5 ± 4.14) (*p* = 0.013).

### 3.4. Entomological Findings

A total of 288 tsetse flies were caught using 60 monoconical traps during the study period. The species identified were *G. tachinoides* and *G. morsitans*. The overall apparent density of tsetse flies was 2.40 FTD. Females constituted 78.82% of the total captured tsetse flies (Table 3).

## 4. Discussions

This study was aimed at assessing the prevalence of trypanosomiasis, risk factors associated with infection rates among the grazing cattle population, and at evaluating the apparent density, distribution, and species of tsetse fly vectors in the Gechi District of the Buno Bedele Zone in western Ethiopia.

This study revealed that bovine trypanosomiasis remains prevalent in the study area. The overall prevalence of bovine trypanosomiasis in the study area was found to be 6.79%. This relatively low level of bovine trypanosomiasis may be attributed to the strategic and frequent tsetse fly control measures implemented by the Bedele Animal Health Research Center (BAHRC), including 1% deltamethrin pour-on treatments and 0.4% deltamethrin impregnated standard targets [9]. However, this rate is higher than those reported by various authors from other tsetse belt areas in Ethiopia. For example, Gebisa et al. [12], Tadesse and Tsegaye [30], Efa [8], and Meharenet et al. [10] reported prevalence rates of 3.4%, 4.4%, 4.4%, and 5.4%, respectively, in the Buno Bedele Zone, Bench Maji Zone, Jimma Arjo District, and districts of western Ethiopia. Alarmingly, over a 4-year time interval, the prevalence of the disease in the Gechi District surged from 1.6% [10] to the current 6.79%, reflecting a 324.4% relative increase. This dramatic rise signals potential ecological, epidemiological, or operational shifts that may be driving transmission dynamics. This aligns with reports of rising trypanosomiasis burdens in southwestern Ethiopia, where agroecological conditions favor tsetse proliferation, particularly in riverine and forest-edge areas characteristic of the landscape of the Gechi District. The other reason could be that emerging resistance to commonly used compounds (diminazene aceturate) might have reduced treatment efficacy [31, 32]. Similarly, lower rates (3.98%) were observed in a study conducted across five local government areas of Kaduna State, Nigeria [33]. These lower prevalence rates suggest that the effectiveness of control measures may vary across areas. Conversely, this result is slightly lower than the findings of Duguma et al. [9], Seyoum et al. [3], Van Den Bossche and Rowlands [34], Lemma et al. [35], and Degneh et al. [36], who reported overall prevalence rates of 9.6% in southwestern Ethiopia, 10.2% in the Gamo Zone, 11.2% in Sayo District, 12.4% in Abeshige District, and 14.1% in Gidami District, respectively. Furthermore, the current finding is lower than earlier reports from other African countries, including Cameroon, with prevalence rates of 9% [37], 10.3% [38], and 29.4% [39], as well as Nigeria, with a recorded infection rate of 15.6% [40]. These higher rates indicate that some areas experience more severe trypanosomiasis challenges, possibly due to differences in agroecology, tsetse fly control measures, or host susceptibility. This balanced evaluation highlights that while the study area's prevalence is moderate, it is higher than that in some areas and lower than that in others. These discrepancies underscore the need for area-specific strategies to effectively control and manage bovine trypanosomiasis.

In this study, among the different species of trypanosomes detected, *T. vivax* was the most prevalent at 71.87%, followed by *T. congolense* at 25%, with the lowest prevalence observed for *T. brucei at* 3.13%. These findings align with those of Mandela et al. [6] and Gebisa et al. [12], who reported the highest proportion of *T. vivax* in northern Uganda and districts of the Buno Bedele Zone of the Oromia Region, respectively. Conversely, Tadesse and Tsegaye [30] reported the highest proportion of *T. congolense* in their study conducted in southwest Ethiopia. Variations in trypanosome prevalence could be influenced by differences in host susceptibility, which encompass intrinsic factors, such as the physiological and nutritional states of the host. The earlier study conducted in the district [10] reported only *T. congolense* and *T. vivax*, with prevalence rates of 1.2% and 0.4%, respectively.

During the study, the prevalence of bovine trypanosomiasis was assessed on the basis of sex, age, and body condition. However, there were no significant associations between the prevalence of the disease and these specific risk factors. This aligns with the findings of a previous study by Haile et al. [41], which also analyzed the same risk factors and reported no significant differences among them. Although not statistically significant, the prevalence of the disease in the peasant associations was greater in Manisa (10.0%) than in Wakale (4.84%), Yabelo (5.59%), and Chitu bosonu (5.88%). This disparity likely stems from higher tsetse fly density in Manisa, driven by its proximity to the Didessa River and the surrounding environment, which provides suitable breeding grounds for the flies and thus elevates disease prevalence.

The PCV can serve as an indicator of the health status of animals. Among the 471 animals examined, 155 (32.91%) had a PCV below the normal range for bovines [34]. Among the 32 positive animals, 18 (56.25%) were anemic. The mean PCV value of aparasitemic animals (25.52%) was greater than that of parasitemic animals (23.13%), which aligns with the findings of Haile et al. [41], who reported a lower mean PCV in parasitemic animals than in aparasitemic animals. This could be because trypanosomiasis causes increased destruction of red blood cells through mechanisms such as erythrophagocytosis, where immune cells consume red blood cells [42] and direct hemolysis caused by the parasite [43]. Additionally, the inflammatory response triggered by infection alters iron metabolism, further impairing the production of new red blood cells and exacerbating anemia [43].

In this study, the entomological findings revealed two species of tsetse flies (*G. tachinoides* and *G. morsitans*) out of the six species reported in Ethiopia [11]. The overall apparent density of *Glossina* species was 2.40 FTD, which is lower than the density reported by Efa [8] and Beshir et al. [13], who reported densities of 6.01 and 18.7 FTDs, respectively. Several factors could either individually or collectively contribute to the observed variation in the density of tsetse flies in different studies and locations, such as variations in seasonal conditions, sampling methods, and the intensity of vector control measures. First, seasonal conditions play a significant role, as changes in temperature, humidity, and rainfall can affect tsetse fly populations [44]. For example, tsetse fly density may increase due to favorable breeding conditions during wet seasons, whereas dry seasons may reduce the number of tsetse flies [44]. Different sampling methods, such as direct catches or baited traps, and specific locations chosen for sampling in different studies, such as proximity to water sources and dense vegetation, can lead to variations in observed tsetse fly densities [45]. Additionally, the intensity of tsetse fly control measures, such as the use of insecticides, traps, or sterile insect techniques, can significantly impact their population, with areas with aggressive control programs often showing lower tsetse fly densities than areas with less intensive efforts [46]. Similarly, this was slightly lower than the tsetse fly density reported in Gechi District in a study conducted 4 years prior to the present study [10]. In their study [10], *G. fuscipes* were found instead of *G. morsitans.*

Among the peasant associations, Manisa had the highest overall density of tsetse flies at 4.65 FTD, which could be responsible for the higher prevalence of infection in that area. A greater percentage of female *Glossina* species (227, 78.8%) were caught than male Glossina species (61.21%). This could be attributed to the longer lifespan of female *Glossina* than that of male *Glossina* [34].

## 5. Conclusion

The present study revealed a 6.79% prevalence of bovine trypanosomiasis in different peasant associations within the Gechi District, with *T. vivax* and *T. congolense* being the dominant trypanosome species identified. Additionally, two tsetse fly species, *G. tachinoides* and *G. morsitans*, were identified. Despite its seemingly low prevalence, continuous implementation of vector and trypanosomiasis control measures in the area is essential to mitigate its impact. Additionally, regular monitoring of both the vector population and the disease prevalence is crucial.

## Figures and Tables

**Table 1:** Prevalence of bovine trypanosomiasis at four peasant associations in Gechi District, western Ethiopia (2022).

**Peasant associations**	**No. examined**	**Positive, ** **n** ** (%)**	**Trypanosome species, ** **n** ** (%)**		
			***T. congolense***	***T. vivax***	***T. brucei***
Chitu bosonu	136	8 (5.88)	3 (2.21)	5 (3.68)	0.00
Manisa	130	13 (10.00)	4 (3.08)	8 (6.15)	1 (0.78)
Yabelo	143	8 (5.59)	1 (0.67)	7 (4.89)	0.00
Wakale	62	3 (4.84)	0.00	3 (4.84)	0.00
Total	471	32 (6.79)	8 (1.69)	23 (4.88)	1 (0.21)

**Table 2:** Logistic regression analysis of risk factors of bovine trypanosomiasis in Gechi District, western Ethiopia (2022).

**Variable (s)**	**Category**	**No. examined**	**Positive, ** **n** ** (%)**	**Crude odds ratio ([COR]: 95% CI)**	**p** ** value**	**Adjusted odds ratio ([AOR]: 95% CI)**	**p** ** value**
Sex	Female	169	12 (7.10)	Ref		Ref	
	Male	302	20 (6.62)	1.08 (0.5–2.3)	0.843	0.9 (0.5–2.2)	0.989

Age	Young	152	9 (5.92)	Ref		Ref	
	Adult	319	23 (7.21)	0.8 (0.4–1.8)	0.604	0.7 (0.3–1.7)	0.464

Body condition	Good	198	13 (6.56)	Ref		Ref	
	Medium	228	13 (5.70)	1.2 (0.5–2.6)	0.710	1.4 (0.6–3.1)	0.468
	Poor	45	6 (13.33)	0.5 (0.2–1.3)	0.135	0.4 (0.2–1.2)	0.107

Peasant association	Manisa	130	13 (10)	Ref		Ref	
	Chitu bosonu	136	8 (5.88)	1.8 (0.7–4.4)	0.218	2.1 (0.8–5.3)	0.133
	Yabelo	143	8 (5.59)	1.9 (0.8–4.7)	0.178	2.6 (0.9–7.2)	0.057
	Wakale	62	3 (4.84)	2.2 (0.6–7.9)	0.236	2.4 (0.6–8.7)	0.196

**Table 3:** Tsetse fly species caught in four peasant associations in Gechi District, western Ethiopia (2022).

**Peasant associations**	**Number of traps**	**Species of fly caught, ** **n** ** (%)**								**Total**	**FTD**
		***G. tachinoides***				***G. morsitans***					
		**Male**	**Female**	**Total**	**FTD**	**Male**	**Female**	**Total**	**FTD**		
Chitu bosonu	12	17	39	56	2.3	4	19	23	1	79	3.29
Manisa	10	16	63	79	4	1	13	14	0.7	93	4.65
Yabelo	25	8	16	24	0.5	0	16	16	0.3	40	0.80
Wakale	13	12	40	52	2	3	21	24	0.9	76	2.92
Total	60	53	158	211	1.76	8	69	77	0.64	288	2.40

## Data Availability

The data that support the findings of this study are available from the corresponding author upon reasonable request.

## Nomenclature

AOR: adjusted odds ratio
BAHRC: Bedele Animal Health Research Center
CI: confidence interval
COR: crude odds ratio
FTD: fly catches per trap per day
*G. morsitans*: *Glossina morsitans*
*G. tachinoides*: *Glossina tachinoides*
PCV: packed cell volume
rpm: revolutions per minute
SPSS: Statistical Package for Social Science
*T. brucei*: *Trypanosoma brucei*
*T. congolense*: *Trypanosoma congolense*
*T. vivax*: *Trypanosoma vivax*

## Supporting Information

Supporting Information Additional supporting information can be found online in the Supporting Information section. File S1: Tsetse fly species found in Ethiopia and their morphological characteristics.
