# Supplementary material for: Assessment of Bovine Trypanosomiasis and Tsetse Fly Density in Gechi District, Western Ethiopia
Source: J Parasitol Res. 2025 Aug 14;2025:5512514. doi: 10.1155/japr/5512514 (PMC12370391; doi:10.1155/japr/5512514)
Supplement: Supporting Information — Additional supporting information can be found online in the Supporting Information section. File S1: Tsetse fly species found in Ethiopia and their morphological characteristics. [file 5512514.f1.docx]

**Supplementary file 1**: Tsetse fly species found in Ethiopia and their morphological characteristics.

| Tsetse fly species | Markings of the back of the abdominal segments | Coloration of the tarsal segments | Size of the fly |
| --- | --- | --- | --- |
| *G. pallidipes* | The middle of the first segment is yellowish and while the middle one is paler. | The last two segments of the tarsus of the front legs are paler. | 8.5-11 millimeter |
| *G.morsitans* | The middle of the first segment is yellowish and other segments are black. | The last segments of the tarsus of the front legs are black. The last two segments of the tarsus of the hind legs are black. | 8-11 millimeter |
| *G. fuscipes* | The middle of the first segment is greyish; all the other segments are black. | All segments of the tarsus of the hind legs are black (black socking). | 8-11 millimeter |
| *G. tachinoides* | The middle of the first segment is yellowish and black bands running across the middle. | All segments of the tarsus of the hind legs are black (black socking). | 6.5-6.9 millimeter |
| *G. longipennis* | Pale reddish brown and no black bands on the back of the abdomen | Stockings reach from the foot to the knee but socks are short. | 11.5-13.5 millimeter |
